# Integration of Expressed Sequence Tag Data Flanking Predicted RNA Secondary Structures Facilitates Novel Non-Coding RNA Discovery

**DOI:** 10.1371/journal.pone.0020561

**Published:** 2011-06-15

**Authors:** Paul M. Krzyzanowski, Feodor D. Price, Enrique M. Muro, Michael A. Rudnicki, Miguel A. Andrade-Navarro

**Affiliations:** 1 Sprott Center for Stem Cell Research, Ottawa Hospital Research Institute, Ottawa, Canada; 2 Max Delbrück Center for Molecular Medicine, Berlin, Germany; 3 Ontario Cancer Institute, Toronto, Canada; University of Illinois, United States of America

## Abstract

Many computational methods have been used to predict novel non-coding RNAs (ncRNAs), but none, to our knowledge, have explicitly investigated the impact of integrating existing cDNA-based Expressed Sequence Tag (EST) data that flank structural RNA predictions. To determine whether flanking EST data can assist in microRNA (miRNA) prediction, we identified genomic sites encoding putative miRNAs by combining functional RNA predictions with flanking ESTs data in a model consistent with miRNAs undergoing cleavage during maturation. In both human and mouse genomes, we observed that the inclusion of flanking ESTs adjacent to and not overlapping predicted miRNAs significantly improved the performance of various methods of miRNA prediction, including direct high-throughput sequencing of small RNA libraries. We analyzed the expression of hundreds of miRNAs predicted to be expressed during myogenic differentiation using a customized microarray and identified several known and predicted myogenic miRNA hairpins. Our results indicate that integrating ESTs flanking structural RNA predictions improves the quality of cleaved miRNA predictions and suggest that this strategy can be used to predict other non-coding RNAs undergoing cleavage during maturation.

## Introduction

Over the past decade, microRNAs (miRNAs) have emerged to be important and powerful factors in most biological processes. miRNAs are small (∼21 nt) species of non-coding RNA (ncRNA) molecules that regulate cognate mRNAs exhibiting sequence complementarity. miRNAs are first transcribed as long primary transcripts (pri-miRNAs) containing hairpin-like structures which are excised by DGCR8/Drosha dependent endonuclease cleavage to form pre-miRNAs [1], [2], [3], which are then exported into the cytoplasm. Pre-miRNAs exported to the cytoplasm are processed by the Dicer ribonuclease to form short dsRNA duplexes, which ultimately supply the RISC complex with mature miRNA strands [4], [5], [6]. Mature miRNAs are thought to coordinate rapid transcriptional transitions by repressing large numbers of mRNA transcripts in parallel [7]. Based on phylogenetic evidence, it is thought that miRNAs arose before vertebrate speciation and play roles to establish tissue identity [8], [9], [10]. During cell growth and differentiation, miRNAs have been shown to function during key transitions between cell states where they facilitate transcriptional reprogramming. Due to their ability to control numerous target transcripts simultaneously, miRNAs have emerged as regulators of gene expression in an increasing number of biological processes involving development and tissue regeneration (e.g. [11], [12], [13], [14]).

Increases in abundance of three major muscle-specific miRNAs (mir-1, mir-133, and mir-206) have been observed during muscle cell differentiation in the mouse, *Drosophila*, and the zebrafish [15], [16]. In mammals, mir-206 can be activated by MyoD expression [17] and remains active in terminally differentiated muscle fibers [18], yet is repressed in developing somites of chicken embryos in response to FGF4 signalling [19]. The induction of myogenic differentiation in C2C12 cells leads to mir-1 upregulation [20], which likely requires Myf5 based on observations of the developing chick myotome [21]. Interestingly, although Myf5 is one of the key regulators of early muscle development, it itself has been shown to be post-transcriptionally regulated by miRNAs in neurons [22], raising the possibility that it is under similar miRNA control in a myogenic context.

These miRNAs are necessary during myogenesis. Completely malformed musculature results when mir-1 is absent during *Drosophila* larvae development [23], and interference with mir-206 in mouse maintains C2C12 cells in a cycling state [24], implying that it is a key requirement for differentiation to proceed. In agreement to this, mir-206 is nearly absent in proliferating porcine satellite cells but is greatly induced during murine C2C12 differentiation [25], [26], suggesting that miRNAs participate in muscle development.

In summary, there is abundant evidence indicating that miRNAs play an important role in specifying the myogenic lineage and it is likely that several undiscovered miRNAs participate in this process. To identify additional miRNAs expressed specifically during myogenic differentiation, we developed and tested a novel strategy of high-throughput tissue-specific miRNA prediction.

Methods employed to predict miRNAs have followed a general theme to identify genomic sites with the ability to produce simple, unbranched RNA hairpin structures and to identify putative RNA hairpins with properties making them distinct from spontaneously arising hairpins [27], [28], [29], with some methods considering conservation data [30], [31] or sequence reads derived from small RNA sequencing [32], [33]. Hybrid approaches to ncRNA prediction attempt to identify novel miRNAs residing within known regions of transcription.

One of the most abundant but underappreciated reservoirs of data to facilitate this work is Expressed Sequence Tag (EST) data previously generated to characterize the transcriptome with over 60 million EST sequences made publicly available in the NCBI dbEST [34]. Several groups have directly examined EST data for ncRNA prediction, attempting to identify novel miRNAs by examining ESTs for hairpin-like structures [35], [36], with others monitoring sites of EST transcription to infer expression of nearby miRNAs [37]. The expression between intronic miRNAs and the protein coding transcripts they lie within is correlated [38], and in fact ESTs have illustrated alternative processing of coding transcripts containing intronic-miRNAs, as in the case of mir-126 within EGFL7 [39].

**Figure 1:**
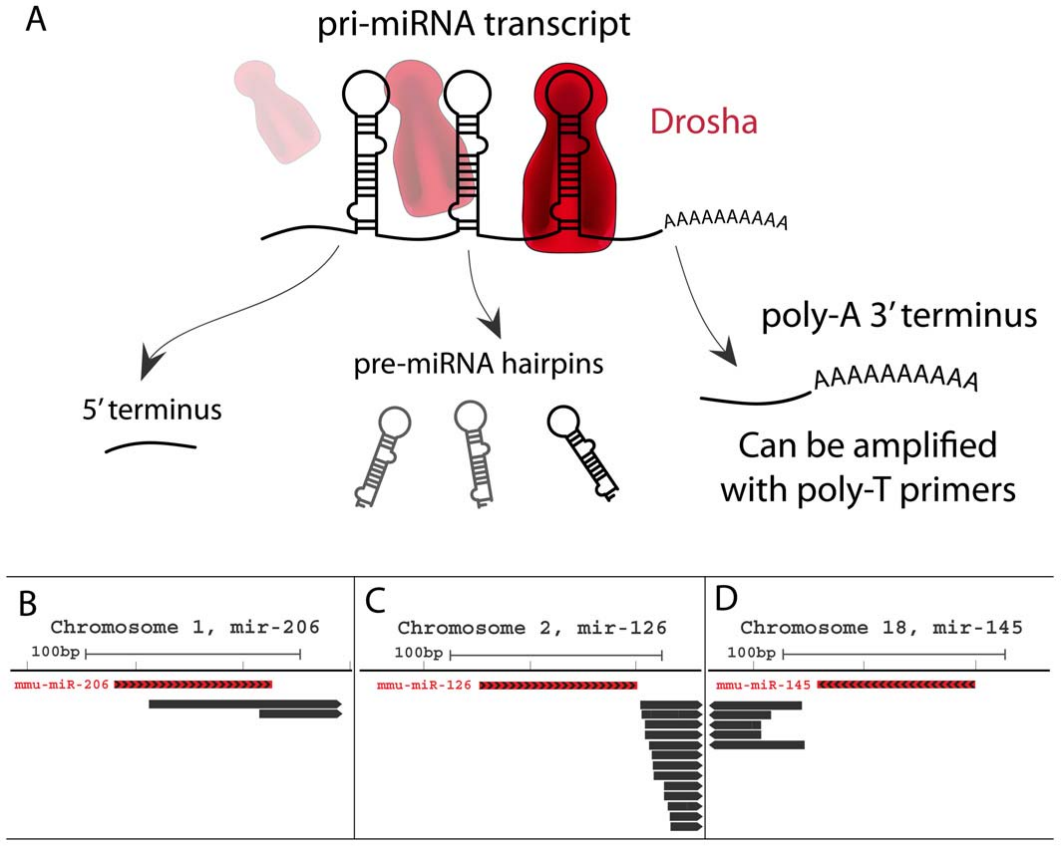
Rationale for miRNA-EST model of prediction. A: Model of miRNA biogenesis and generation of EST evidence of miRNA expression. RNA polymerase II transcribes the primary miRNA transcript (pri-miRNA), which is cleaved by the Drosha-DGCR8 complex. Cleaved pre-miRNAs are further processed, while intervening fragments are degraded. The remaining polyadenylated 3′ fragments of the pri-miRNA may be amplified by polyT primers commonly used in the generation of EST libraries. B, C, D: Examples of known miRNAs with ESTs in proximity to their 3′ terminus (Mouse genome, mm9).

miRNA precursor molecules are polyadenylated [40], [41], [42], [43], [44], and can be successfully amplified using oligo-dT primers [45]. In fact, the usage of poly-T primers in EST library construction allows for observation of the 3′ ends of pri-miRNA cleavage products of miRNA biogenesis, with reverse transcription ending at the 5′ terminus of a miRNA hairpin (Figure 1A). As many examples of this effect are seen such as loci of murine mir-145, mir-126 and mir-206 (Figure 1B), we investigated the utility of adding EST data in a miRNA prediction pipeline.

To this end, we developed a methodology that combines techniques from ncRNA prediction with EST sequences. Our novel reinterpretation of preexisting public EST data identifies traces of miRNAs and provides expression predictions from tissue-specific EST libraries. This method constitutes a de facto novel contribution to techniques of predicting tissue-specific ncRNAs.

## Methods

### Source data

We used mm8, mm9 and hg18 releases of the mouse and human genomes. Four-way conservation data used in RNAz included human (hg18), rat (rn4), dog (canFam2) and mouse (mm8 or mm9, where appropriate). Where required, miRNA, EST annotations, and multiZ annotations were obtained from the appropriate releases of the UCSC genome annotation datasets [46].

### Generating ncRNA predictions

ncRNA predictions were generated using three methods: RNALfold [47], RNAz [48], and alignment of high-throughput sequencing fragments derived from a small RNA library provided in GEO record GSM314552 [33]. For details of the implementation of each of these methods, see the supporting information.

### Integrating EST data with known miRNAs

To identify and visualize relationships between miRNAs and ESTs, 3′ or 5′ coordinates of miRNA annotations on human and mouse genomes (hg18 and mm8, respectively) were identified and 2 kb sequence windows centered on this location were retrieved. To compute a normalized score of EST occupancy in this region, coordinates of EST termini mapping within each sequence window were identified and a score for each site was incremented by 1/*n*, where *n* equals the number of ESTs mapped to the miRNA associated sequence window. Scores for all miRNAs were aggregated, binned in 10 nt windows, and plotted. Significance scores for peaks observed in each genome assembly were determined by bootstrapping. Briefly, for each miRNA window, window coordinates were shuffled to random locations on the same chromosome and EST termini were identified in ±1 kb genomic windows. This procedure was replicated 10,000 times. EST accumulation was scored as described above, and the scores for each genome assembly and test (upstream/downstream of miRNA) were aggregated. The number of times a replicate generated a central peak in excess of the observations was counted and used to determine a P-value.

### Generating EST supported miRNA predictions

To screen candidate miRNA hairpins, we filtered hairpin structures based on their normalized ΔG value and the distance to a downstream EST terminus, as true miRNAs were observed to have lower than average normalized free energy (NFE) than the entire set of genomic hairpins computed with RNALfold. We thus examined the effect of filtering predicted structures based on precision/recall values computed using NFEs between 0 and −1 kcal/mol and EST proximities between 0 and 200 nt (See Figure S1). We retained predicted hairpin structures exhibiting a maximum NFE of −0.44 kcal/mol and with one or more EST termini within the structure, permitting the generation of mature miRNA and up to 14 nt downstream of the 3′ end. Predictions generated by RNAz were considered on either strand as RNAz did not preserve information regarding strandedness. For mapped Illumina sequencing fragments, which approximate mature miRNA sequences, not pre-miRNA hairpins, we determined the maximal distance between sequencing fragments and ESTs. We examined the distances between both 5′ and 3′ termini of known mature miRNAs and the ends of their corresponding pre-miRNAs (Figure S2). As we were not able to retrieve the strandedness of the mature miRNA sequences from the dataset due to its experimental design, we assumed that the mature miRNAs may reside in either arm. In the result, we were forced to use the greater of the two ‘d’ values which considers the possibility that the predicted mature miRNA resides in the 5′ arm of the pre-miRNA. The mean of the larger distances was 51 nt, which was used as a cutoff for considering sequence fragment/EST intersections.

### Statistics

Enrichment statistics for EST supported miRNA predictions were determined by shuffling candidate miRNA annotations to random locations within the same chromosome and strand. Candidate miRNAs were then predicted by intersecting EST data according to the methods described above.

Precision and recall of miRNA predictions were computed using as positives a set of known miRNAs or the subset of those supported by transcript data if such data were being used in prediction. MiRNA predictions were considered supported if they had EST annotations within 14 nt for those predicted computationally with RNALfold or RNAz, or within 51 nt for those predicted using high-throughput sequencing. For high-throughput sequencing data, a known miRNA was considered as transcript-supported if there was at least one Illumina sequencing fragment mapped within the known miRNA structure. For miRNA predictions generated by RNALfold and RNAz, a known miRNA was considered as supported if at least 75% of the nucleotides in the predicted hairpin and miRNA structure overlapped.

### miRNA predictions

13267 miRNA predictions were generated using RNA hairpin predictions in the mouse genome (mm8). Supporting dbEST annotations (retrieved September 9, 2007) were integrated with RNA structures as explained above to add source tissue predictions. A summary of miRNA predictions generated for mouse (mm9) is in Table S1. For immediate validation purposes, miRNAs with predicted expression in muscle (32), myotube (88), embryo (822), and embryonic stem cell (674) were selected.

### Design and analysis of a miRNA microarray

To detect expression of putative miRNAs, a custom oligonucleotide tiling microarray with 60mer probes (Agilent) was designed to tile over selected known and predicted miRNA hairpins with probes overlapping by 20 nt. Control probe sequences including positive and negative controls for known miRNAs, other ncRNAs, randomized negative control sequences, and sequences designed against known protein coding genes were designed (Table S2). In addition to the numbers of muscle, myotube, embryonic, and embryonic stem cell miRNA predictions (32, 88, 822, and 674 respectively) referenced in the previous paragraph, space permitted the addition of candidates with normalized hairpin free energies that met relaxed cutoff values. RNA preparation and microarray normalization were done according to the manufacturer's recommended protocols.

### miRNA target predictions

miRNA predictions were calculated using the TargetScan protocol [49]. The algorithm was replicated locally with custom scripts, which produce results highly correlated with published miRNA-target scores (r>0.99; Data not shown).

### Cell cultures

The mouse ES cell line J1 was maintained on a layer of irradiated MEFs (DR4) in DMEM high glucose medium (Invitrogen) supplemented with 15% fetal bovine serum (Hyclone), 1× non essential amino acids, 1× sodium pyruvate, 1× Glutamax, β-Mercaptoethanol, 1× Pen/Strep (Invitrogen), and 1000U ESGRO (chemicon) at 37°C and 5% CO_2_. Prior to RNA isolation ES cells were trypsinized and preplated for 1 hr on 0.1% gelatin plates to remove the majority of MEFs. Mouse C2C12 cells and HEK293T cells were maintained in DMEM high glucose medium supplemented with 10% fetal bovine serum (Hyclone) and 1× Pen/Strep at 37°C and 5% CO_2_. For differentiation, C2C12 cells were grown to confluence and subsequently cultured in DMEM high glucose with 2% horse serum (Hyclone) for 3 days prior to RNA isolation. R1, C2C12, and HEK293T cells are available from ATCC as ATCC Numbers SCRC-1011, CRL-1772, and CRL-11268, respectively.

### Northern Blotting

To validate putative miRNAs by Northern blotting, total RNA was isolated using the mirVana kit according to the manufacturer's instructions (Ambion). 5 ug of total RNA was resolved with 12.5% urea-polyacrylamide gels and electroblotted onto Hybond NX membranes (GE). 60mer 5′ biotinilated oligonucleotides complementary to short hairpin RNA sequences were used as probes (Table S3). Membranes were developed using the light shift chemiluminescent kit with a streptavidin horse radish peroxidase secondary antibody according to the manufacturer's instructions (Pierce). Membranes were exposed to Biomax MR film (Kodak) for visualization.

## Results

### EST termini are significantly associated with 3′ termini of miRNAs

**Figure 2:**
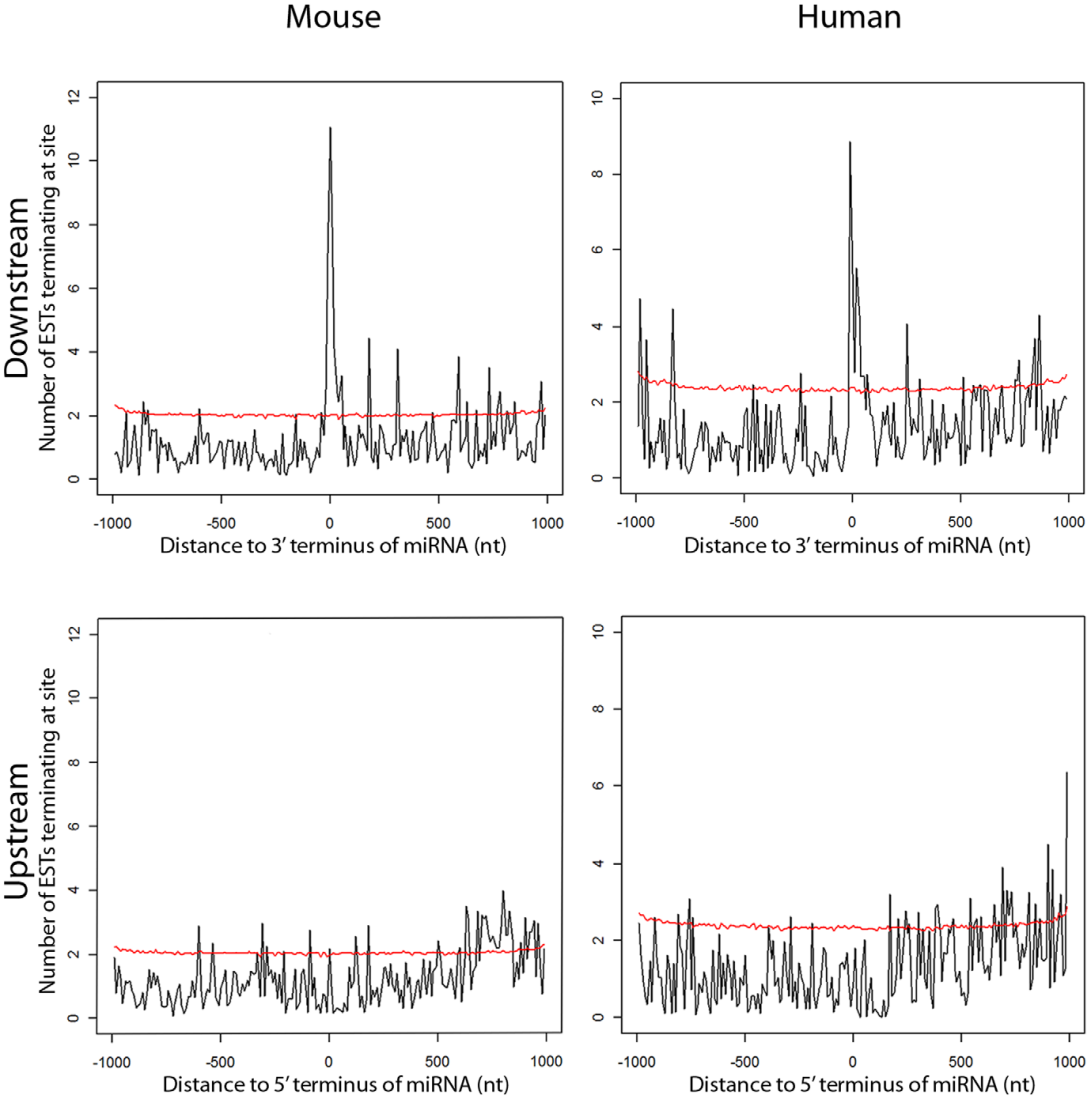
EST termini associate with 3**′** end of annotated miRNA in mouse and human genomes. Y-axes measure the normalized density of observed EST termini within each genomic window surrounding known miRNAs. Top: Accumulation of EST termini adjacent to 3′ ends of known miRNAs in the mouse (mm8) and human (hg18) genomes. Bottom: This effect is not observed at the 5′ ends. The background signal obtained with randomly distributed ESTs and miRNAs is shown (red lines).

To determine whether ESTs could be used as a general data source in novel miRNA prediction, we examined whether a trend of association exists between the EST termini around known miRNAs in human and mouse. We observed a distinct clustering of EST termini in sequence regions aligned with the 3′ ends of known miRNA in the mouse and human genomes, respectively (Figure 2). By comparison, no clustering of EST termini was observed when examining the 5′ ends of known miRNAs indicating the validity of our rationale for interpreting EST data (Figure 1A).

One important issue in genome wide screens is ascertaining the degree of background signal present. To estimate the extent of this, the ncRNA-EST analysis was repeated numerous times using randomly shuffled miRNA positions. The results in Figure 2 show that 3′ termini of miRNAs are associated with ESTs above expected background levels. This result is independent of the genome assembly version (Figure S3). Together, these observations support our model of concurrent miRNA and EST generation presented in Figure 1. With this information, we inferred that miRNA predictions might be generated by identifying genomic regions potentially generating structured RNA with corresponding EST evidence in proximity of their 3′ terminus.

### EST data enrich the quality of microRNA predictions

To investigate whether improvments in miRNA predictions can be attained with this strategy, we considered whether potentially structured RNA regions demonstrating supporting EST evidence would benefit miRNA prediction pipelines, considering three different methods to identify miRNA candidates: simple hairpins generated by RNALfold [47], conserved RNA structures (RNAz) [48], and mapped high-throughput sequence fragments residing in hairpins.

RNALfold is a method of generating structured RNA predictions that identifies energetically stable RNA structures within subregions of very long sequences. After calibrating settings to discard predicted RNA structures unlikely to be miRNAs (See Methods and Figure S4 for details), we obtained approximately 3.3 million predicted hairpins in the mouse genome (mm9). In this set of RNA hairpins, known miRNAs were found to have lower than average Normalized Free Energy (NFE) (in agreement to [50]). This was reproduced through three different NFE computations, the most significant (P<9×10^−80^) when using the nucleotides contained within the duplexed region excluding those in the single stranded tails and terminal RNA loop (See Figure S5). We therefore refined this set of 3.3 million predicted hairpins by retaining those with both lower than average NFE (under −0.44 kcal/mol) and ESTs within 14 nt of the 3′ terminus (see Methods for determination of these values), yielding 41,347 candidates.

The association between ESTs and predicted structured RNA regions is significantly higher than expected if one were to assume a random distribution of genomic annotations (P<0.0001; Table 1). The rates of known miRNA prediction were examined with and without including known ESTs in the protocol for three different methods of predicting ncRNAs (Table 2). Using the set of RNALfold generated hairpin predictions, known miRNAs were predicted with a recall rate of almost 85% but a precision rate of only 0.01%. Upon incorporation of EST data, 81 out of 91 (89%) annotated miRNAs with ESTs within 14 nt were recalled by using this criteria while the precision rate increased to 0.20%, likely due to the loss of randomly arising hairpins not associated with EST evidence. Therefore, by considering known miRNA annotations recoverable with EST data, we effectively increased the recall rate by approximately 5% while simultaneously increasing the precision by 20 fold.

**Table 1:** ncRNA-EST associations are significantly higher than expected by chance.

Genome	Object type	n objects	n ESTs	Intersecting @14 nt	Bootstrap mean	S/N Ratio	Bootstrap P-value
mm8	RNALfold^1^	3299070	4561569	41347	33182	1.24	<10−4
	RNAz^2^	79094	4561569	17245	4176	4.13	<10−4
	Illumina^3^	3442	4561569	112	25	4.48	<10−4
mm9	RNALfold^1^	3340947	4364783	40872	32496	1.26	<10−4
	RNAz^2^	62057	4364783	12617	3039	4.15	<10−4
	Illumina^3^	3446	4364783	88	18	4.89	<10−4

1 Hairpins predicted using RNALfold.

2 Hairpins predicted using RNAz.

3 Mature miRNA sequences predicted using Illumina high-throughput sequencing data.

**Table 2:** Considering EST annotations improves rates of miRNA prediction.

		mm9					
		Illumina^1^		RNALfold^2^		RNAz^3^	
		w/o ESTs	w/ESTs	w/o ESTs	w/ESTs	w/o ESTs	w/ESTs
A	Raw sequencing objects	3446	3446	3340947	3340947	62057	62057
B	Sequencing objects considered	3446	540	3340947	40782	62057	12617
C	Unique miRNAs detected	348	108	393	81	144	35
D	Total miRNA predictable	463	132	463	91	463	91
	Precision (C/B)	10.10%	20.00%	0.01%	0.20%	0.23%	0.28%
	Recall (C/D)	75.16%	81.82%	84.88%	89.01%	31.10%	38.46%

1 Mature miRNA sequences predicted using Illumina high-throughput sequencing data.

2 Hairpins predicted using RNALfold.

3 Hairpins predicted using RNAz.

In a similar fashion, we generated predictions of structured RNAs exhibiting interspecies conservation between mouse, human, dog and rat using RNAz, a method that considers genomic alignments when generating candidate ncRNAs [48]. RNAz based ncRNA predictions were again highly significant (Table 1). However, the RNAz based results generated a higher Signal-to-Noise ratio than raw hairpins generated by RNALfold, presumably because interspecies conservation eliminated many potential false positives from the set of putative ncRNA hairpins. Applying EST data to RNAz predictions produced a modest increase in both precision by 22% (0.23% to 0.28%) and recall by 24% (31% to 38%) (Table 2). The lower recall rate observed with the RNAz implementation may be due to either some known miRNAs being located in genomic regions with lower conservation scores or to some miRNAs being species specific.

To investigate the utility of other types of transcript data for analysis of small RNA sequence libraries, we examined the influence on miRNA predictions of using published data derived from an embryonic stem cell library generated with Illumina sequencing technology [33]. Sequence fragments deposited in GEO (Accession: GSM314552) were aligned to the mouse genome, identifying 4239 small (∼21 nt) sequence windows from which RNA expression was captured. After restricting these sequences to windows predicted to be within putative RNA hairpins, 3446 potential mature miRNA sequences with experimental support remained (see Methods for details). In a similar fashion to the other analyses discussed above, known miRNAs were intersected with EST windows, but a distance of 51 nt between sequencing fragments and ESTs was used to account for sequenced miRNAs corresponding to mature miRNAs, due to mature miRNA windows being nested within pre-miRNAs which are predicted computationally (Figure S2). Without EST data, the analysis yielded rates of recall and precision of 75.2% and 10.1%, respectively. When putative small RNA sequence windows were intersected with EST annotations to retain locations with both forms of experimental support, we observed the rates of recall and precision rise to 81.8% and 20.0% (Table 2).

Together, the results from the above three benchmarks strongly argue that improvements in the prediction rates of ncRNAs, and specifically ncRNAs undergoing processing or cleavage, such as miRNAs, can be attained if future ncRNA prediction algorithms incorporate EST data into their protocols.

### Myoblast expressed miRNA-like hairpins are identified by a miRNA microarray

Beyond indicating the extent of transcription, records in the dbEST database provide valuable information describing the tissues, cell types, and conditions in which ESTs were observed. We reasoned that these data could be used to predict tissue-specific context in which our predicted miRNA could be expressed.

To demonstrate that this method could be used to produce tissue-specific results of biological significance we predicted novel myogenic miRNAs. Cross-referencing information from genomic sites containing predicted RNA hairpins and EST evidence derived from muscle or myoblast tissues, we identified 196 potential sites of miRNAs expressed in myogenic cell types. An overview of various numbers of predictions produced in muscle, myoblast, or other tissues is shown in Table S1. We used a tiling microarray to examine the expression of each genomic site containing predicted miRNA hairpins with likely expression in differentiating myoblasts or mES cells.

The custom tiling microarray was designed to detect sequences for each putative miRNA hairpin site, along with multiple control sequences corresponding to known miRNAs. In total, 13840 probes tiling over putative miRNAs were designed. RNA from differentiating C2C12 myoblasts and J1 embryonic stem (ES) cells was isolated and hybridized to the microarrays in duplicate, for a total of eight hybridizations. Correlations between replicates are shown in Table S4.

After data normalization and analysis, 148 probe sets with >2 fold enrichment in muscle RNA versus ES derived RNA were identified, including muscle control probe sets designed against known muscle miRNAs mir-206, mir-133a/b (Figure S6), as well as the let-7 family miRNAs. In contrast, control miRNAs derived from the mir-291 cluster were strongly expressed in the RNA derived from ES cells, as expected. Taken together, the microarray data showed correct and robust reciprocal expression of major control miRNAs in each cell type, suggesting that numerous miRNA-like hairpins are differentially expressed in differentiating myoblasts.

### Known miRNAs expressed in differentiating myoblast cultures

Several known miRNAs that were captured by our prediction method were also detected as differentially expressed in myoblasts compared to ES cells. Several putative miRNA probe sets correspond to known miRNAs mir-24-2, mir-351, mir 26a-2, mir-92-2 and mir-7-2. According to these probes, miRNAs mir-24-2, mir-351, and mir-26a-2 were potentially upregulated in differentiating myoblasts, while mir-92-2 and mir-7-2 loci were downregulated (Table 3). Individual probes in each probe set exhibiting differential signal were manually verified to overlap with the sites of their corresponding mature miRNAs, with the exception of mir-7-2, suggesting that differential expression of mature miRNAs was observed. The changes in expression for probes at the mir-7-2 locus are likely due to non-specific hybridization.

**Table 3:** Hairpins with miRNA overlap exhibiting differential expression on microarray.

Hairpin ID	Fold Increase		miRNA	Location
	C2C12	ES		
hp1643988	141.56	-	mir-24-2	chr8:87098910-87099035
hp3202291	108.27	-	mir-351	chrX:49297888-49297981
hp907540	94.12	-	mir-26a-2	chr10:126398456-126398589
hp1980840	-	1.52	mir-92-2	chrX:48986474-48986552
hp85655	-	1.72	mir-7-2	chr7:78761795-78761882

To better understand the context by which these microarray signals could have arisen, the genomic contexts of these candidates were examined. Of highest relevance to the observed expression in myoblasts, the miRNA hairpin mapping to mir-26a-2 was found to be intronic to a long splice variant of *Ctdsp2* (carboxy-terminal domain, RNA polymerase II, polypeptide A, small phosphatase 2); it appears that a shorter *Ctdsp2* variant would be produced if this miRNA were excised from the longer transcript. *Ctdsp2* is able to induce mesodermal lineage commitment in a *Xenopus* system [51], while mir-26a-2 is known to be expressed during myogenesis, where it represses Enhancer of Zeste (Ezh2) transcript levels [52].

mir-351 is potentially part of a miRNA cluster with mir-503 and mir-322 lying within 2 kb upstream, with numerous ESTs mapped in this region supporting this possibility. However, no contiguous regions of expression appear to span the genomic region defined by these three miRNAs. mir-24-2 is the downstream most member of miRNA cluster containing mir-27a and mir-23a. This cluster is downstream from *Zswim4*, an uncharacterized zinc finger containing protein.

### Prediction of targets for known miRNAs

To identify potential targets for the miRNAs expressed at higher levels in differentiating myoblasts, the TargetScan method of identifying miRNA targets was used. Potential target transcripts, which mature sequences for mir-24-2, mir-26a, and mir-351 might be repressing, were predicted and rather than considering targets individually, scores were combined multiplicatively to identify potential transcripts that might be synergistically repressed by all three (Table 4), a method that penalizes targets where one or more scores are abnormally low. Immediately striking was the high rank for potential targeting of the Dystrophin (*Dmd*) transcript.

**Table 4:** Top 10 predicted targets for synergistic repression by mir-24, mir-26a, and mir-351.

		Targetscan Scores		
Symbol	Description	miR-24	miR-26a	miR-351
Plod2	Procollagen-lysine,2-oxoglutarate 5-dioxygenase 2 Precursor	−0.3234	−0.7219	−0.2086
Dmd	Dystrophin	−0.352	−0.2867	−0.4483
Tbc1d30	TBC1 domain family member 30	−0.4755	−0.5532	−0.1713
Ret	Proto-oncogene tyrosine-protein kinase receptor ret Precursor (C-ret)	−0.3827	−0.5018	−0.1895
4732454E20Rik	Protein FAM26D	−0.7775	−0.3364	−0.1225
Irf4	Interferon regulatory factor 4	−0.1166	−0.516	−0.5252
Papola	Poly(A) polymerase alpha	−0.5424	−0.229	−0.2491
Zfp697	Zinc finger protein 697	−0.492	−0.3602	−0.1722
Slc25a35	Solute carrier family 25 member 35	−0.3222	−0.2178	−0.4084
St8sia4	CMP-N-acetylneuraminate-poly-alpha-2,8-sialyltransferase	−0.3275	−0.335	−0.2319

Dystrophin is transcribed from a large genomic region (∼3 megabases), contains 79 exons, and undergoes numerous alternative splicing patterns [53]. As the second best target, Dystrophin was targeted most robustly by mir-351 (TargetScan score = −0.45). Upon closer inspection, the Dystrophin 3′UTR targeted by all three miRNAs was a longer Dmd transcript (ENSMUST00000114000) that terminates approximately 1 kb beyond the main transcription stop site, and which has a single EST as evidence of transcription (CK385805) in the current version of dbEST. Despite the low number of ESTs associated with this Ensembl transcript, it is not possible to conclude that the *in vivo* expression of this longer Dmd transcript is proportionally low. In contrast, Dystrophin's shorter 3′UTR is targeted only by mature mir-26a (Data not shown). As different Dystrophin splice variants are expressed in different tissues, with the 3′ region of the transcript undergoing alternative splicing to produce tissue-specific transcripts in brain, cardiac and muscle fibers [53], it is possible that miRNAs may be involved to enforce transcription of some Dmd isoforms over others in certain contexts.

### Novel miRNA-like hairpins are expressed in differentiating myoblasts

**Figure 3:**
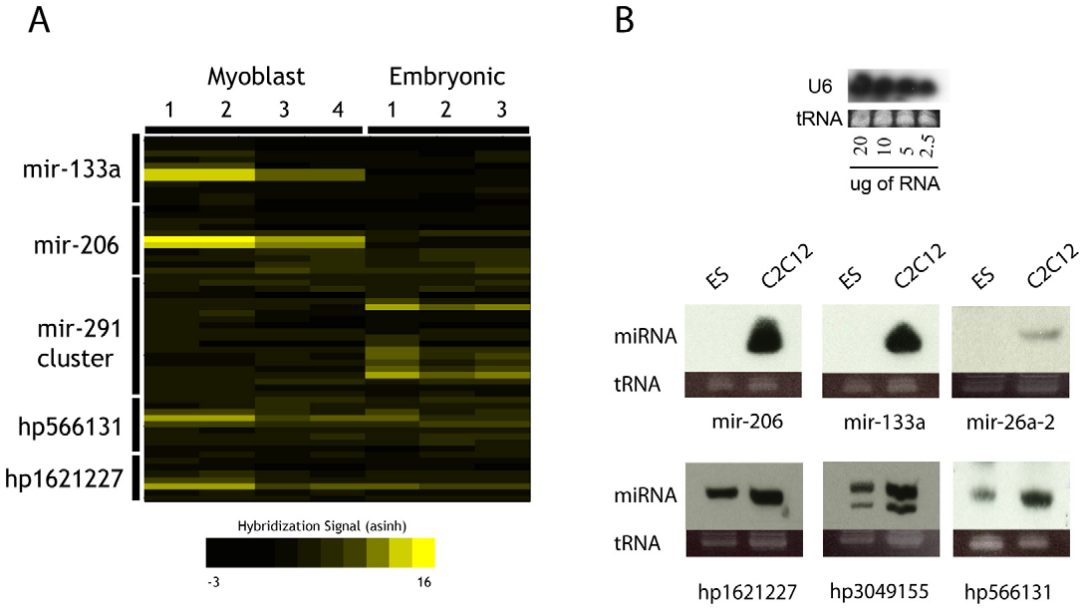
Detection of known and predicted miRNAs. A: Detection of control miRNAs via microarray in C2C12 and mES RNA samples. Numbers for each sample type indicate replicates. B: Northern blotting of several known and predicted miRNAs exhibiting enrichment in differentiating C2C12 myoblasts.

Several putative ncRNA hairpins with strong expression scores were selected to determine whether the expression and molecular size of the RNAs were consistent with miRNA presence. Northern blotting was used to visualize the size of RNA species responsible for microarray signals, using the respective microarray probe sequences. Several of the selected probe sets indeed detected expression of small RNAs in the ∼20–30 nt size range, suggesting that mature miRNAs were being generated (Figure 3A). Microarray data detected three predicted ncRNA hairpins at higher levels in myoblast — hp566131, hp1621227, and hp30491555 — and Northern blotting supplied information regarding their size and also confirmed their differential expression (Figure 3B). Identifiers with hp- prefixes denote RNA hairpins predicted in the course of this analysis. These results indicate that the microarray signals were due to small RNA species, likely derived from predicted miRNA hairpins, which are expressed in myogenic cells undergoing differentiation at higher levels than in embryonic stem cells.

## Discussion

We showed that addition of EST data in ncRNA prediction protocols can significantly improve prediction rates, using miRNAs as a representative class of ncRNAs. In doing this, we show that taking into account predicted behaviours of one type of transcript, inferences can be made as to what might be observed in data produced for entirely disparate purposes. In this study, we combined the features of EST generation (priming poly-A transcript ends) with knowledge of the RNA cleavage activity during miRNA biogenesis, to develop an approach that uses EST records and information about predicted RNA structures to predict miRNAs as a class of hairpin derived ncRNAs. Our results show that future development of non-coding gene prediction strategies should combine secondary sources of biological data with primary sources of information such as sequence composition, motifs, and predicted RNA secondary structures when knowledge of RNA transcript behaviour facilitiates the development of an integrative model. We emphasize that our method is not an alternative miRNA prediction method but rather contributes an analytical step that can improve the quality of existing miRNA prediction strategies. For this reason, we explicitly showed that integrating non-overlapping EST and miRNA annotations enable improving miRNA prediction methods.

We furthermore expect that including EST data in ncRNA prediction strategies is a generalizable approach to other models of ncRNAs undergoing cleavage during their production or function. We can illustrate this latter point with the Clec2d 3′UTR, which has been reported to contain a self-cleaving hammerhead ribozyme [54]: upon manual inspection, we observed that the ribozyme cleavage site lies in close proximity to a cluster of ESTs (Data not shown), suggesting that novel ribozymes might be predicted by considering co-occurrence of ESTs and covariation models for specific RNA families.

It must be noted that adding EST evidence, as with the addition of any data, imposes a constraint on the analysis such that some miRNA predictions are called as false negatives. This trade-off must be considered. However, the significant increases of precision and recall for miRNA predictions that pass the integration step suggest that follow up experiments using these first tier candidates can experience lower false discovery rates. Nonetheless, those interested can still investigate second tier candidates but now with the additional knowledge that false discovery rates in these predictions will be lower due to the lack of EST evidence to support them.

On a practical level, we also illustrated how pre-existing data can produce compelling improvements in the performance of current high-throughput sequencing technologies. The increased precision and recall rates demonstrated in Table 2 illustrate how rates of ncRNA validation can be doubled when considering the results of the small RNA high-throughput sequence data. The combination of these data can justify small scale validation trials when genome wide analysis is not being pursued, as the doubling of precision with small RNA sequencing data to support miRNA predictions imply that manual validation attempts can be halved for similar outcomes. This combination is also one where two separate experimentally generated sets of data supported computational predictions and yielded the most dramatic improvements in precision rates, clearly highlighting the benefit of working within a single model (miRNA biogenesis) and saturating it with as much biological data as available. The improved results from this example also hint at potential synergistic effects when using independent sources of high-throughput data (i.e. compensating for technique-specific biases), which may become addressable on a broad scale as mutually compatible data sets accumulate.

A second practical contribution of ESTs towards ncRNA prediction is in supplying probable tissues of expression in which validation of ncRNAs can be pursued, for example when a specific tissue is of interest. Integrating data this way can also simplify experimental designs by minimizing sample size requirements, limiting resources consumed in pursuing specific questions.

Next-generation sequencing is emerging as a successor to EST library sequencing with RNA-seq becoming commonly available. However, as far as we know, no database of next-generation sequence transcript data exists that is comparable to dbEST. The wide distribution of conditions sampled in dbEST and the standardized format of annotations facilitate the discovery of transcripts that possibly occur in very particular conditions such as the ones we have presented here. The analysis of deep-sequencing data in an EST-like fashion still involves curation of primary sequence reads, assembly of short reads into contigs, and finally alignment to genomic positions, each step potentially requiring manual tuning. Should a database of deep-sequencing transcript data similar to dbEST be generated in the future, we have no doubt that our method will be successfully applied to it.

### On known miRNAs expressed in myoblast samples

Using a custom miRNA microarray allowed us to verify the performance of our method by capturing known miRNAs expressed in myoblast samples. We observed that three known miRNAs, mir24-2, mir-351, and mir-26a-2 were upregulated in myoblasts and showed robust expression. Expressed at 141 times the level observed in the embryonic stem cell sample, mir-24-2 was the most strongly overexpressed mature miRNA, and has previously been captured during myogenic differentiation [55]. mir-24-2 lies in a cluster alongside miRNAs mir-23a and mir-27a upstream of a gene encoding ZSWIM4, an uncharacterized zinc-finger containing protein. This miRNA cluster is activated in other cellular differentiation related contexts with similar effects, such as during the formation of multiple blood cell lineages during hematopoiesis [56] and during hepatic stem cell differentiation [57], being potentially associated with regulation of cell fate choice. While superficially it seems that the mir-24 containing cluster is a generalized differentiation controlling locus, mir-24 has been observed to be specifically expressed in porcine satellite cells in a reciprocal fashion to mir-206 [25]. The placement of mir-24 in a rare myogenic population may hint at a role in regulating stem cell entry into differentiation, possibly by the control of timing or proportions of cells entering the process. Similarly, mir-26a-2 has previously been demonstrated to function in repressing the polycomb group gene Enhancer of Zeste (*Ezh2*) during myogenesis [52]. Ezh2 is known to be involved in myotome development, where it functions to inhibit progress through differentiation [58]. Remarkably, *Ezh2* is also active in an analogous system – epidermal differentiation – where it has recently been shown to contribute towards control of differentiation rates throughout multistage epidermal shedding [59]. Thus, the mir-26a-2/Ezh2 system may function as another control mechanism to balance cell differentiation rates and speed.

Finally, mir-351 was upregulated in myoblasts at levels 108-fold over ES. In the mouse, mir-351 lies in a region of EST activity which includes mir-503, mir-322, and an uncharacterized transcript, which appears to undergo splicing (dbEST identifier AK021262). Taken together, these elements may represent a multipart transcript, and interestingly four additional miRNA coding sites (mir-542, mir-450a-2, mir-450a-1, and mir-450b) reside nearby, which do not have any evidence of being transcribed based on known dbEST sequence records. The function of mir-351 and this region is unknown, but mir-351 has been observed in SAGE tags derived from embryonic heart [60]. With this data and given the density of miRNA features and transcriptional activity, it may be worthwhile to investigate this region for potential involvement in muscle tissues.

We were interested in identifying predicted co-repression of target transcripts by all three mature miRNAs observed in C2C12s (mir24-2, mir-351, and mir-26a-2), reasoning that they might act in concert to exert effects on transcripts comparable to much more highly expressed miRNAs that are solitary actors such as mir-206. Considering combined miRNA repression scores, the best target identified was *Plod2*, encoding a collagen cross-linking enzyme with two isoforms exhibiting tissue specific expression in adult, with strongest protein expression observed in muscle tissues [61], [62]. *Plod2* is an attractive target as the integrity of muscle tissue is strongly dependent on extracellular matrix components, and particular collagen subtypes are associated with myoblasts in different stages of proliferation [63]. What is especially enigmatic of *Plod2* is that both isoforms are transcribed in muscle yet only the longer isoform undergoes translation [62]. If this is a case of the shorter isoform being specifically repressed by miRNAs, it would be in contrast to the model of gene dysregulation by miRNA binding site loss via 3′UTR truncation, which has been used to explain dysregulation of oncogenic mRNAs [64]. Given the expression of *Plod2* isoforms in muscle and their differential translation, combined with potential targeting by three known miRNAs with expression in muscle, exploring miRNA mediated control of *Plod2* expression may be promising.

The second best target identified was Dystrophin (*Dmd*), encoding a myotubular structural protein critically linked to the manifestation of Duchenne Muscular Dystrophy [65]. Dystrophin is not expressed during early differentiation of myoblasts until fusion into myotubes has begun [66]. Closer inspection of the Dmd 3′UTR regions identified two transcriptional termination sites, with the variant targeted by all three miRNAs being longer than the major termination region covered robustly by known ESTs. If this interaction is true, it may hint at a case where active miRNAs are specifically responsible in eliminating Dmd transcripts created from abnormal termination events, a role that has been reported in cases where alternative isoforms are specifically targeted to control their abundance levels [67], [68], [69]. In all, our results concerning mir24-2, mir-351, and mir-26a-2 upregulation in myoblasts represent added evidence for miRNA based regulation of myogenic genes and suggest that deeper involvement of ncRNAs in myogenesis will be revealed.

### General conclusion

We demonstrate that considering EST data in ncRNA prediction algorithms improves results when predicting tissue-specific miRNAs, and propose that EST data be included in future ncRNA prediction pipelines, especially of those that are polyadenylated and cleaved. In addition, we identified several known miRNAs with expression in differentiating myoblasts, representing potential participants of differentiation during this process. Understanding the role of these miRNAs and ncRNAs may provide insight into how endogenous capacities for muscle regeneration are determined.

## Supporting Information

Figure S1 **Calibration of filter based on Normalized Free Energy and miRNA-EST distances.** Cutoffs for Normalized Free Energy (NFE) and miRNA-EST distance were calibrated to filter the set of hairpins generated from the genomewide scan of RNALfold. Precision/Recall curves shown for each miRNA-EST distance (*d*) cutoff, with individual points corresponding to NFE cutoffs at each value of *d* (Points for *d* = 14 indicated in black with NFE values decreasing from 0 to −1 counterclockwise). Circled point corresponds to *d* = 14 and NFE = −0.44 kcal/mol. NFE values range from 0 to −1 kcal/mol and miRNA to EST distances range from 0 nt to 200 nt.
(TIF)

Figure S2 **Determination of mature miRNA placement within pre-miRNA windows.** (A) 553 mature miRNA sequences were mapped within their parent pre-miRNA hairpin annotations and distances between termini were measured. d_1_ and d_2_ correspond to the shorter and longer distances, respectively. (B) Distributions of the values. The mean of the d_1_ and d_2_ are shown (12.7 nt and 51.3 nt, respectively).
(TIF)

Figure S3 **Distributions of EST peaks near miRNAs are similar across genome versions.** Distributions of miRNA-ESTs are highly similar across different versions of the mouse genome (mm8 and mm9). EST occupancy surrounding miRNAs was calculated as described in Methods.
(TIF)

Figure S4 **Calibration of real-time RNALfold filter parameters.** Precision and recall values were calculated by applying RNALfold on genomic DNA sequences centered on known miRNAs and filtering resultant RNA structures. Filtering parameters used were: hairpin size (Degree of blue shading); hairpin NFE (Degree of red shading); and RNALfold window size (Proportional to point size). Parameters used in this study and corresponding precision and recall are shown with a white X. For computing precision/recall, miRNA-hairpin annotations had a minimum overlap of 80%.
(TIF)

Figure S5 **Distributions of Normalized Free Energies in sets of miRNAs and set of genome-wide predictions.** Three distributions are shown: The red, black, and blue distributions correspond to values derived by normalizing the total minimum free energy (MFE) by the number of nucleotides in the hairpin sections as highlighted in the inset figure.
(TIF)

Figure S6 **Control miRNAs are expressed correctly on microarray.** Heatmap showing correct expression of control miRNA probe sets for muscle (mir-133a and mir-206) and ES cell control (mir-293 containing cluster). Probe sets were composed of multiple probes tiling over each pre-miRNA hairpin sequence, and only probes overlapping mature miRNA sequences yielded expression signals.
(TIF)

Table S1 **Overview of predicted miRNA hairpins in different tissues.** 13952 unique putative miRNA hairpins were annotated in the mouse genome (mm9), based on predictions generated from RNA structures with supporting EST annotations. Counts above do not correspond to this value due to some miRNA predictions having ESTs derived from multiple sources, and sources with under 10 predictions being excluded due to space limitations.
(XLS)

Table S2 **Table of RNA control sequences included on the miRNA microarray.** Individual miRNAs with specific roles included where possible. In cases of clustered miRNAs where one member of a cluster was identified with a specific function (e.g. ES miRNA), additional members of the cluster were tiled as well.
(XLS)

Table S3 **Probe sequences used in Northern blotting.**
(XLS)

Table S4 **Pearson correlation between replicates of miRNA tiling microarray.** All samples were performed in duplicate.
(XLS)
