# Supplementary figures and images for: Integration of Expressed Sequence Tag Data Flanking Predicted RNA Secondary Structures Facilitates Novel Non-Coding RNA Discovery
Source: PLoS One. 2011 Jun 15;6(6):e20561. doi: 10.1371/journal.pone.0020561 (PMC3115948; doi:10.1371/journal.pone.0020561)

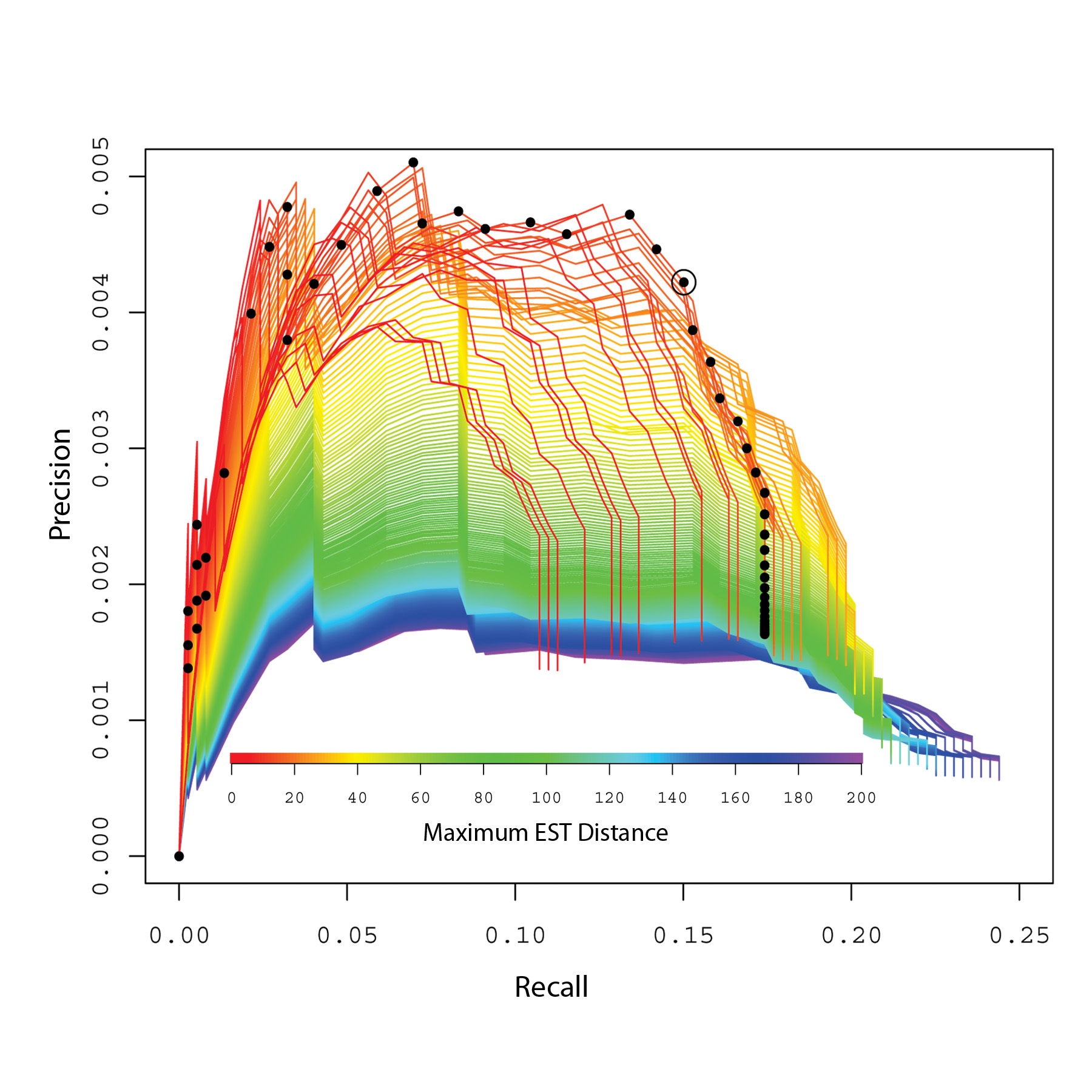

Supplement: Figure S1 — Calibration of filter based on Normalized Free Energy and miRNA-EST distances. Cutoffs for Normalized Free Energy (NFE) and miRNA-EST distance were calibrated to filter the set of hairpins generated from the genomewide scan of RNALfold. Precision/Recall curves shown for each miRNA-EST distance (d) cutoff, with individual points corresponding to NFE cutoffs at each value of d (Points for d = 14 indicated in black with NFE values decreasing from 0 to −1 counterclockwise). Circled point corresponds to d = 14 and NFE = −0.44 kcal/mol. NFE values range from 0 to −1 kcal/mol and miRNA to EST distances range from 0 nt to 200 nt. (TIF) [file pone.0020561.s001.tif]

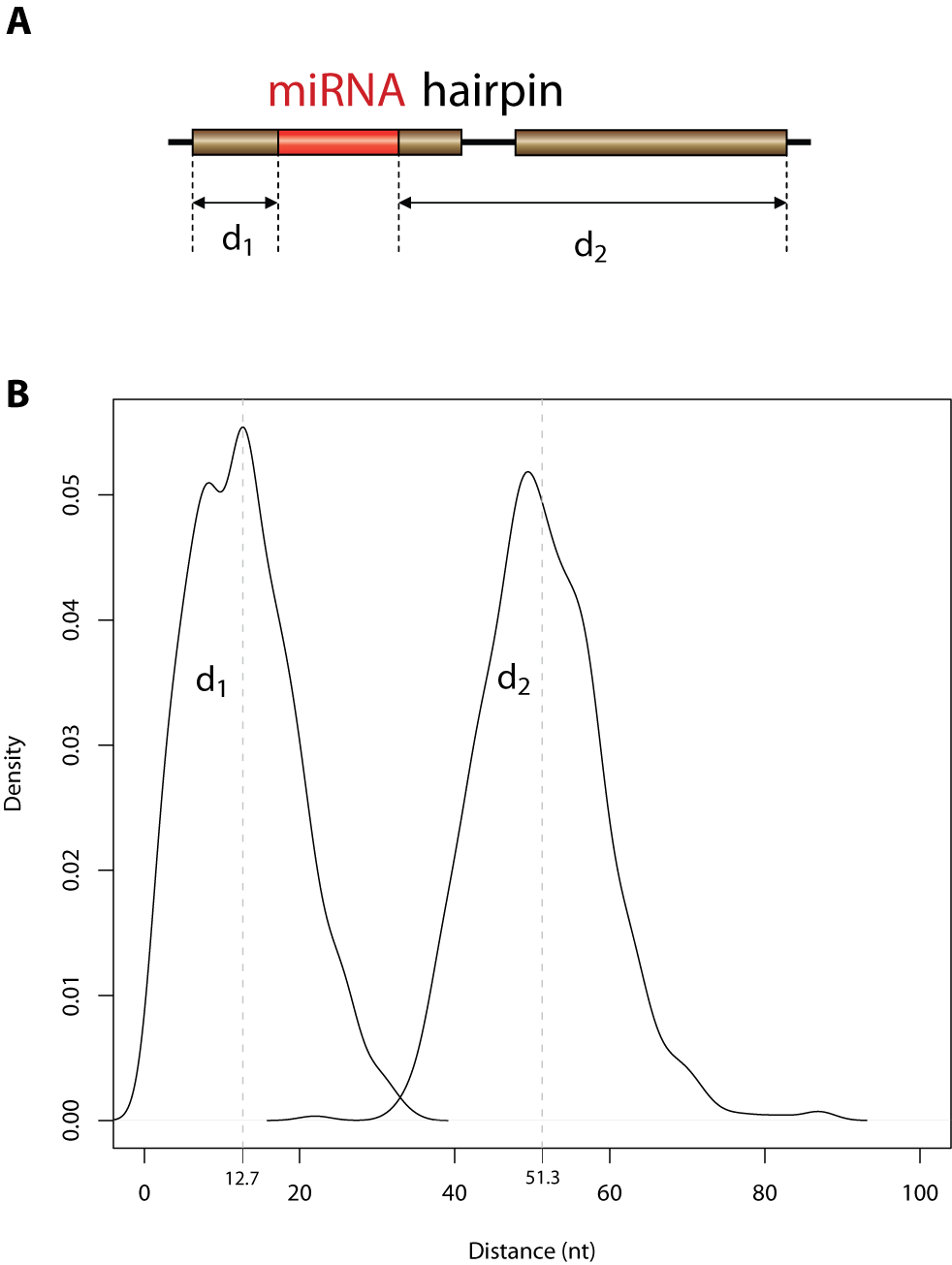

Supplement: Figure S2 — Determination of mature miRNA placement within pre-miRNA windows. (A) 553 mature miRNA sequences were mapped within their parent pre-miRNA hairpin annotations and distances between termini were measured. d1 and d2 correspond to the shorter and longer distances, respectively. (B) Distributions of the values. The mean of the d1 and d2 are shown (12.7 nt and 51.3 nt, respectively). (TIF) [file pone.0020561.s002.tif]

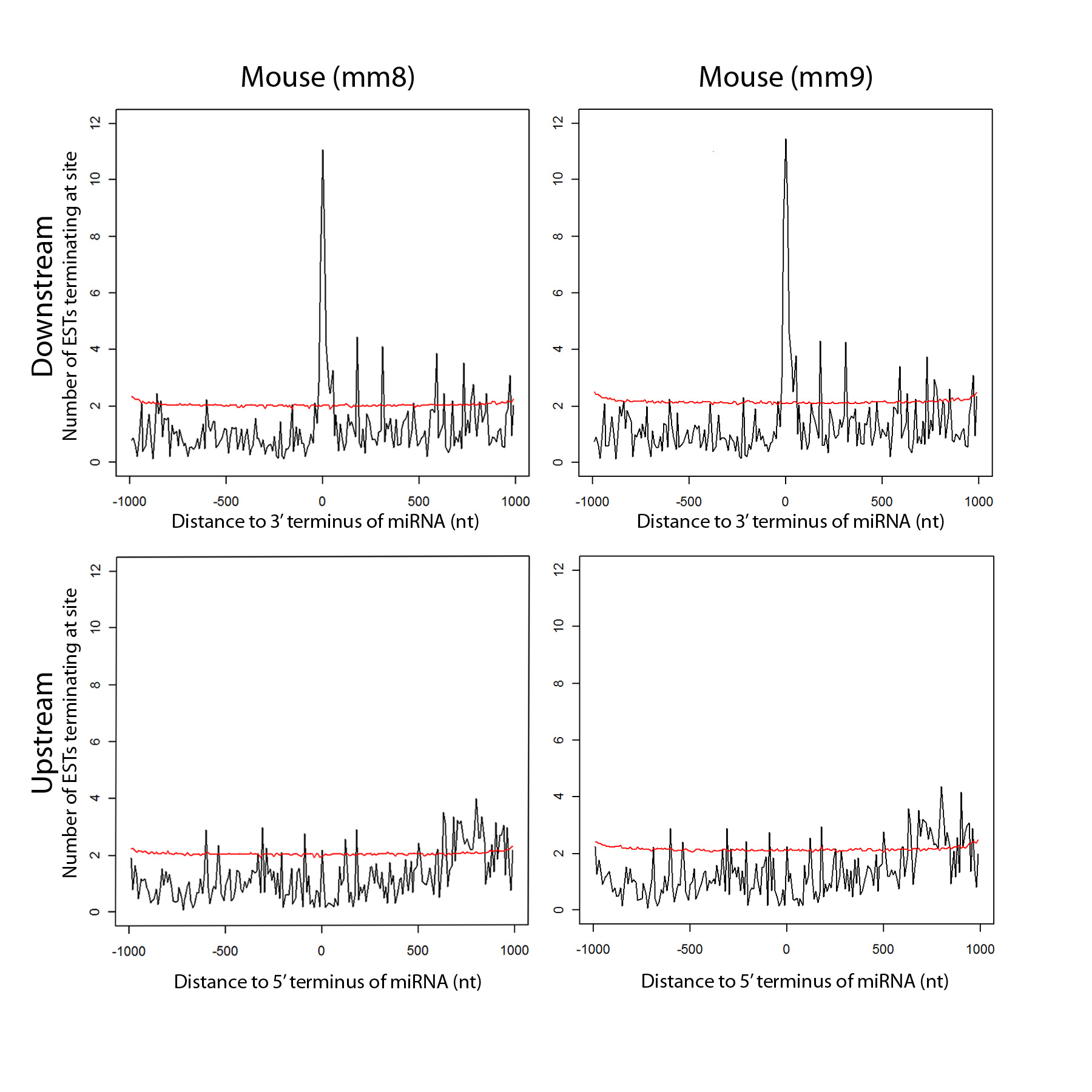

Supplement: Figure S3 — Distributions of EST peaks near miRNAs are similar across genome versions. Distributions of miRNA-ESTs are highly similar across different versions of the mouse genome (mm8 and mm9). EST occupancy surrounding miRNAs was calculated as described in Methods. (TIF) [file pone.0020561.s003.tif]

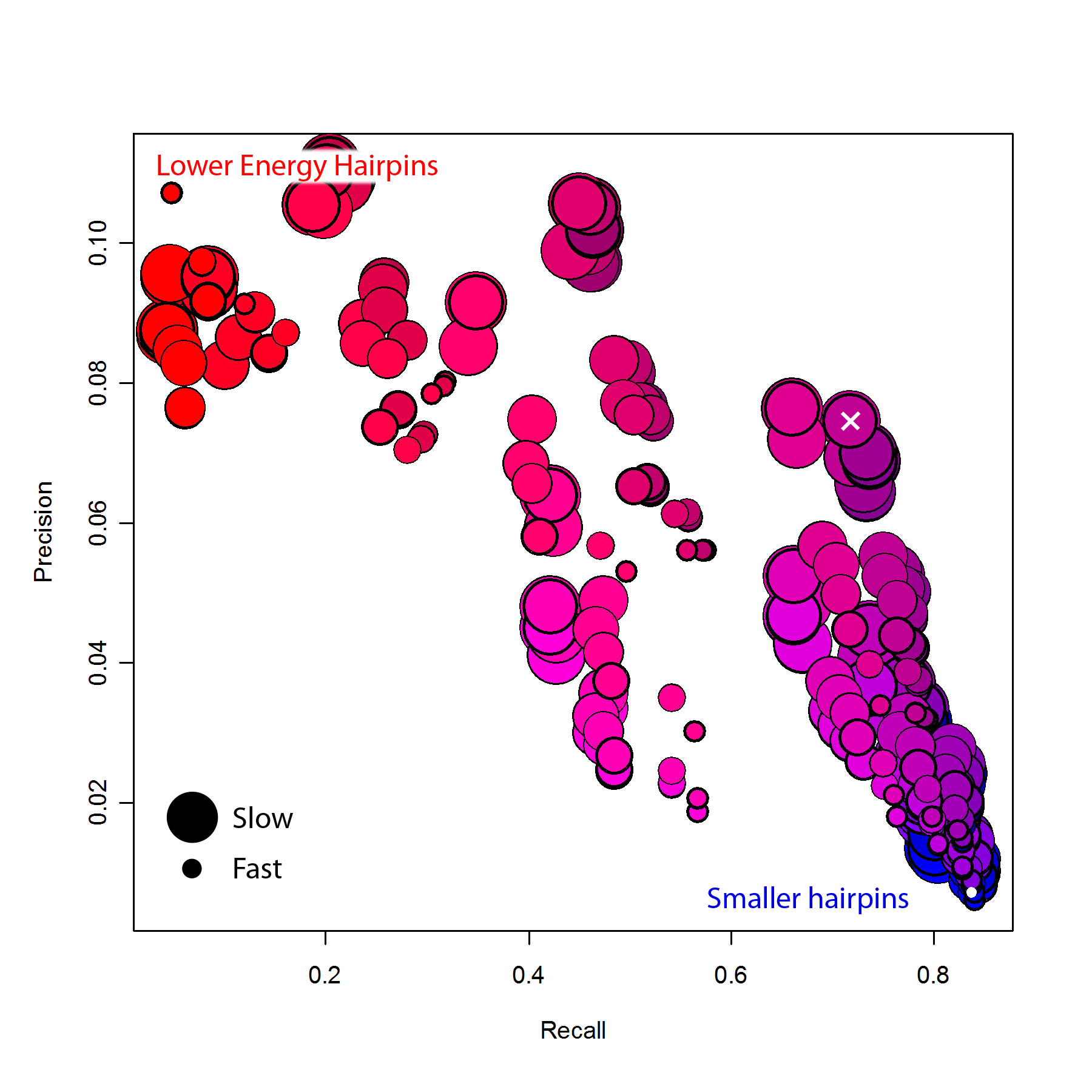

Supplement: Figure S4 — Calibration of real-time RNALfold filter parameters. Precision and recall values were calculated by applying RNALfold on genomic DNA sequences centered on known miRNAs and filtering resultant RNA structures. Filtering parameters used were: hairpin size (Degree of blue shading); hairpin NFE (Degree of red shading); and RNALfold window size (Proportional to point size). Parameters used in this study and corresponding precision and recall are shown with a white X. For computing precision/recall, miRNA-hairpin annotations had a minimum overlap of 80%. (TIF) [file pone.0020561.s004.tif]

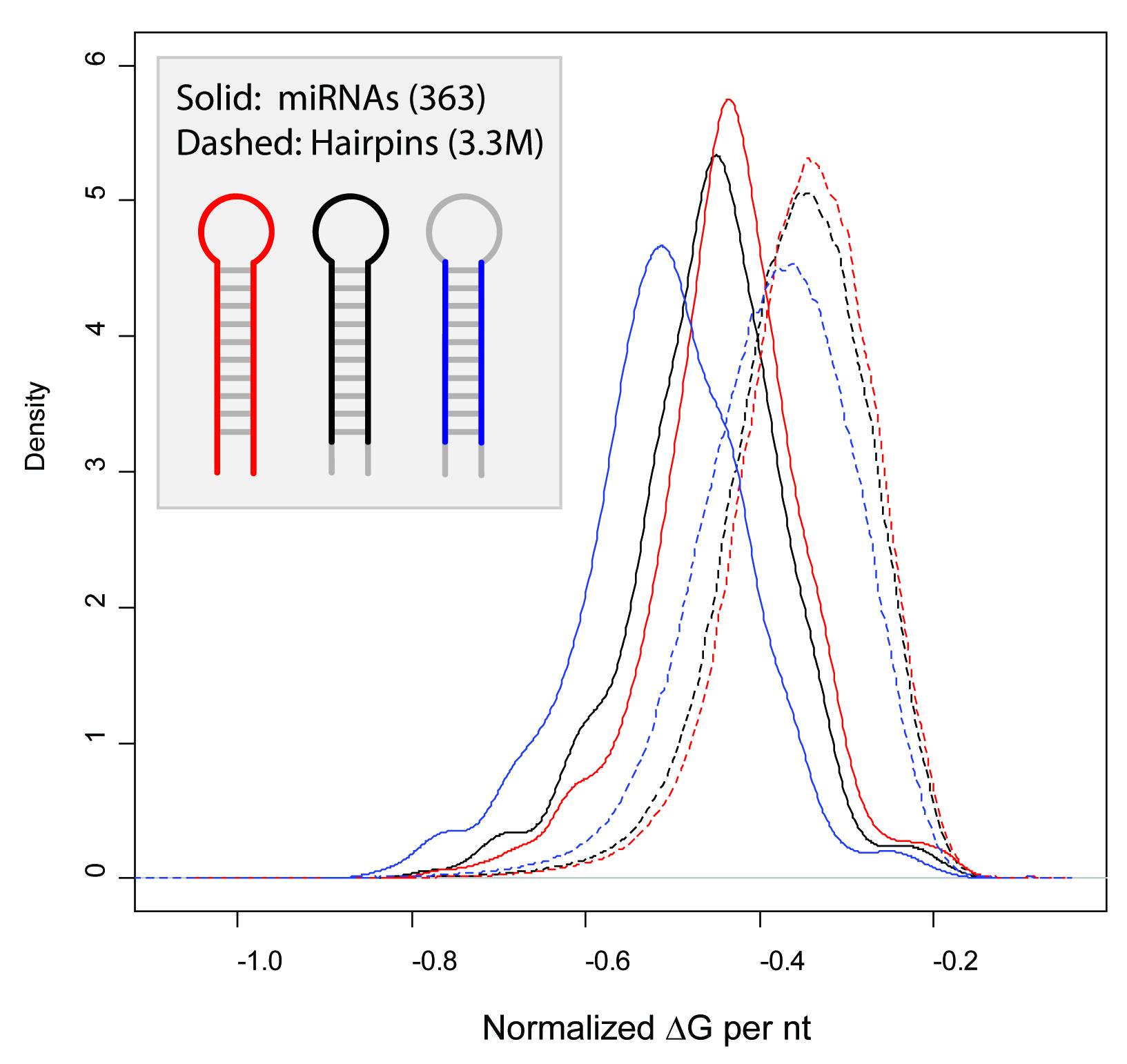

Supplement: Figure S5 — Distributions of Normalized Free Energies in sets of miRNAs and set of genome-wide predictions. Three distributions are shown: The red, black, and blue distributions correspond to values derived by normalizing the total minimum free energy (MFE) by the number of nucleotides in the hairpin sections as highlighted in the inset figure. (TIF) [file pone.0020561.s005.tif]

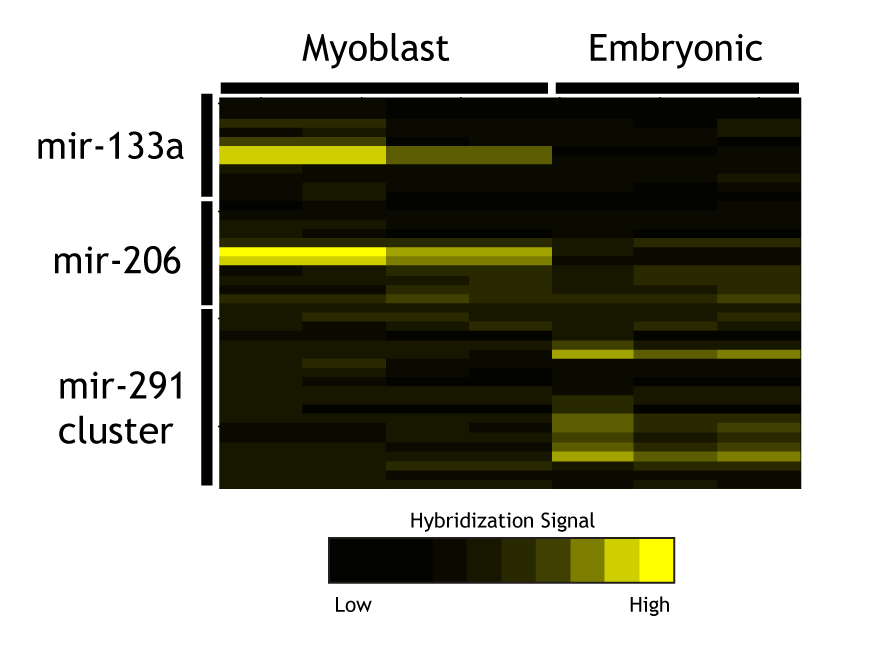

Supplement: Figure S6 — Control miRNAs are expressed correctly on microarray. Heatmap showing correct expression of control miRNA probe sets for muscle (mir-133a and mir-206) and ES cell control (mir-293 containing cluster). Probe sets were composed of multiple probes tiling over each pre-miRNA hairpin sequence, and only probes overlapping mature miRNA sequences yielded expression signals. (TIF) [file pone.0020561.s006.tif]
